# NucleoSeeker—precision filtering of RNA databases to curate high-quality datasets

**DOI:** 10.1093/nargab/lqaf021

**Published:** 2025-03-18

**Authors:** Utkarsh Upadhyay, Fabrizio Pucci, Julian Herold, Alexander Schug

**Affiliations:** John von Neumann Institute for Computing, Jülich Supercomputing Centre, 52428 Jülich, Germany; Computational Biology and Bioinformatics, Université Libre de Bruxelles, 1050 Brussels, Belgium; Interuniversity Institute of Bioinformatics, 1050 Brussels, Belgium; Scientific Computing Center, Karlsruhe Institute for Technology, 76344 Karlsruhe, Germany; John von Neumann Institute for Computing, Jülich Supercomputing Centre, 52428 Jülich, Germany; Department of Biology, University of Duisburg-Essen, D-45141 Essen, Germany

## Abstract

The structural prediction of biomolecules via computational methods complements the often involved wet-lab experiments. Unlike protein structure prediction, RNA structure prediction remains a significant challenge in bioinformatics, primarily due to the scarcity of annotated RNA structure data and its varying quality. Many methods have used this limited data to train deep learning models but redundancy, data leakage and bad data quality hampers their performance. In this work, we present NucleoSeeker, a tool designed to curate high-quality, tailored datasets from the Protein Data Bank (PDB) database. It is a unified framework that combines multiple tools and streamlines an otherwise complicated process of data curation. It offers multiple filters at structure, sequence, and annotation levels, giving researchers full control over data curation. Further, we present several use cases. In particular, we demonstrate how NucleoSeeker allows the creation of a nonredundant RNA structure dataset to assess AlphaFold3’s performance for RNA structure prediction. This demonstrates NucleoSeeker’s effectiveness in curating valuable nonredundant tailored datasets to both train novel and judge existing methods. NucleoSeeker is very easy to use, highly flexible, and can significantly increase the quality of RNA structure datasets.

## Introduction

Deep learning (DL) technology has given a significant boost to scientific research by providing powerful tools for data analysis, pattern recognition and prediction [1]. It strongly impacted the computational structural biology community enabling the recent breakthroughs such as AlphaFold [2] providing a massive improvement in both speed and accuracy for protein structure prediction.

Prior methods based on statistical inference such as direct coupling analysis (DCA) [3] gave a glimpse at the value hidden within the evolution of biomolecular sequences, enabling the statistical inference of spatial adjacencies to guide structure prediction tools [4, 5]. Transformer networks as used by models such as AlphaFold [2] leverage the information of protein evolution as found in sequence data to derive structural information. While these approaches are highly successful [6], they require abundant training data.

**Figure 1.**
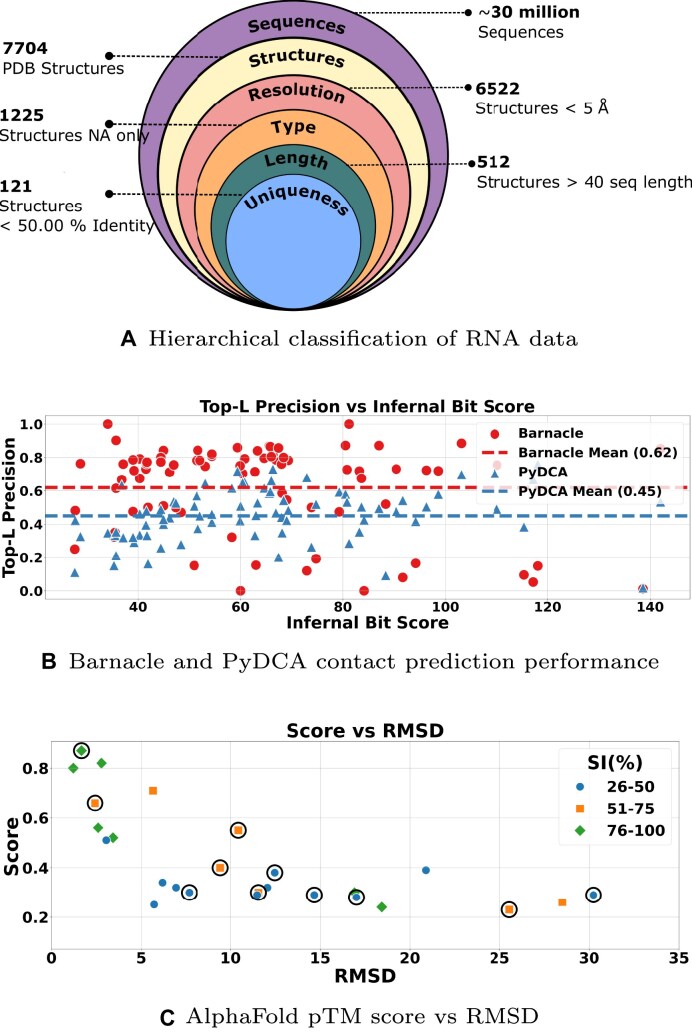
(**A**) Hierarchical classification of RNA data: from 30 million sequences to 121 unique structures. This nested diagram illustrates the progressive filtering of RNA information, showcasing the rarity of well-characterized, unique structures among the vast sea of known sequences. Each layer represents increasingly stringent criteria. (**B**) Barnacle and PyDCA contact prediction performance: Top-L precision of two RNA contact prediction tools, Barnacle (red circles) and PyDCA (blue triangles), for the $\mathcal {D_{C}}$ structures as a function of their Infernal Bit Score with the corresponding RFAM families (*x*-axis). (**C**) AlphaFold pTM score versus RMSD: AlphaFold pTM score as a function of the Root Mean Square Deviation (RMSD) between AlphaFold3 predictions and experimental structures in Å, categorized by sequence identity (SI%) levels.

Thus, scarcity of data prohibits the direct transfer of these methods to other biomolecules, e.g. RNA. Moreover, the available RNA structural data suffers not only from its limited size but also from high redundancy and low data quality. Specifically, the current version of the Protein Data Bank (PDB) [7] contains a large number of highly similar RNA structures, structures with poor resolution, a significant number of hybrids (protein/RNA, DNA/RNA, and others), and a considerable proportion of very short sequences (fewer than 20 residues) (see Fig. 1A and Supplementary Fig. S1 in Supplementary Data).

Such properties often cause DL models to overfit and generalize poorly. Assessing DL models is also challenging because data leakage, stemming from improper splits between training and test sets, can lead to an overestimation of the model’s performance [8, 9].

Furthermore, reproducibility also becomes impaired. Experiments like RNA-Puzzles [10] and CASP [11], where computational algorithms have to blindly predict RNA structures, provide a valid evaluation method. However, results [11] suggest that due to the above-mentioned problems, DL models currently perform worse than physics-based approaches in RNA structure prediction task.

The development of curated datasets for training and testing models is essential to addressing these issues. For instance, following strict filtering processes and manual curation as in [12, 13] can be highly effective. Here, we introduce NucleoSeeker, an easy-to-use software that provides extensive flexibility and control for curating RNA datasets from structures deposited in the PDB database in a fully automated manner.

## Materials and methods

NucleoSekeer is a python library that can be directly used as a command-line tool with limited dependencies (i.e. Biopython, Pandas, Numpy, and Requests). It handles downloading and applying filters to create a dataset.

### Dataset access

Initially, no structures are downloaded from the RCSB PDB database. Instead, we first use the Search API of the PDB to retrieve all IDs for a specified structure determination method and a given polymer entry type. These IDs are then processed through a GraphQL query, which fetches predefined attributes, such as the experimental method used, resolution, and many more for each structure (see Section S1 of Supplementary Data for details). This API-based approach ensures that our tool generates the most up-to-date dataset without requiring any code modifications. This module yields a data frame (DF) with all requested IDs and their corresponding attributes.

To refine the dataset, we use three different kinds of filters in our software that allow the users to specify their requirements for various levels from the individual chain to multiple structures; all the filter modules in the package are also available as standalone modules.

### Dataset creator

The results of the filtering operations are combined to generate a dataset of RNA structure. To further increase the diversity of the dataset we use cmscan utility of Infernal [14] software. It searches the sequences against the covariance models present in the RFAM database [15]. This annotates each entry in the dataset with its corresponding RFAM family, based on the best hit, while selecting only the lower *E*-value entry per RFAM family to further reduce redundancy. Users have the option to specify the *E*-value threshold for the RNA family hits; this specifies the statistical significance of the result (refer to the user guide of Infernal [14] for more details). A lower *E*-value indicates a more significant result, effectively controlling the strictness of the family search.

The output of NucleoSeeker consists of a list of RNA chains along with corresponding information, such as RFAM classification, PDB code and the corresponding number of chains, structure resolution, experimental method used, and year of release. Note that, in the case of complexes, only the RNA chains that meet all the specified criteria are selected.

### Filtering mechanisms

In our filtering approach, we follow the hierarchy used by the RCSB PDB database to organize structures. More in detail, we use the three common levels ENTRY, ENTITY, and INSTANCE, which form the basis of the different modules.

Here, we will briefly describe the functionality of the different modules and how they integrate to generate the final dataset. An exhaustive list of arguments and parameters for each module can be found in Supplementary Table S1. Note, that the word Polymer is used in multiple parameters across modules, and each of them carries a different meaning (see Supplementary Table S1).

#### Metadata

We use this module to apply filters based on the metadata stored in the DF. This offers a comprehensive set of filters for structural attributes, e.g. users can filter structures based on the experimental methods used for determination, such as X-ray diffraction, nuclear magnetic resonance, or Cryo-EM, ensuring that only structures determined by these techniques are included. The module also offers a resolution threshold filter, allowing users to exclude structures with resolutions outside a specified value. Similarly, structures can be filtered by their release year, allowing the inclusion of only those resolved before a specific year or within a particular range of years. Furthermore, the module supports selection based on polymer entity types, enabling users to customize their datasets by focusing on specific polymers or excluding polymer complexes. Additionally, the keyword filter provides the ability to include or exclude structures based on specific terms, offering further refinement of the dataset to meet users’ requirements. Finally, there is also an option to filter structures based on RNA sub-types such as non-coding RNA (ncRNA), ribosomal RNA (rRNA) etc. to enable specific research applications. An exhaustive list of supported RNA sub-types is provided in the Supplementary Table S1.

#### Individual structure

The IndividualStructure module analyzes the composition of structures for further processing, i.e. it parses individual files. It examines each structure’s polymer type, nucleotide or residue integrity, and the length of each chain. This module can function independently to download and verify PDB files based on specified criteria when given a PDB ID. Utilizing a PDB parser, it extracts and analyzes structural information, allowing users to specify parameters such as the desired polymer instance type and sequence length to filter out chains that do not meet these criteria. This module enables the easy removal of short sequences and the extraction of chains containing only RNA.

#### Structure comparison

This filter level combines our filtered DF from the Metadata filter and the IndividualStructure filter by applying the latter to each structure in the DF. We also integrate sequence alignment tools, such as Clustal Omega [16] and Emboss [17], to filter structures based on sequence identity (SI). If the SI between two structures exceeds a certain threshold, We select the structure with the highest resolution to minimize redundancy in the dataset while ensuring good overall structure resolution.

A SI matrix is created using the specified alignment tools. However, since EMBOSS processes sequences in pairs, impacting performance, it is generally recommended to use Clustal Omega for most applications.

These filters, whether used individually or collectively, provide an unprecedented level of control and flexibility in curating datasets from the PDB database. Consequently, researchers can create more targeted and refined datasets, significantly enhancing the accuracy and reliability of their RNA structure prediction protocols.

## Results

Well-curated and non-redundant datasets serve two important goals: they improve training outcomes and are crucial for assessing method performance. Here, we show that NucleoSeeker can be used to attain this goal. In particular, we highlight two examples where our tool can create datasets for assessing RNA contact prediction methods and evaluating AlphaFold3 [18] performance on RNA structure prediction. The manual curation of such datasets is time-consuming and error-prone and can lead to nonsystematic biases. We show the ease of curating such datasets using NucleoSeeker and believe it can be used to prepare datasets for machine learning algorithms in a similar way.

### Use-case 1: automated RNA structures curation for assessing contact prediction

We used NucleoSeeker to create a well-curated and non-redundant dataset starting from all the 7704 RNA structures available in the PDB database (accessed in July 2024). We selected only RNA structures resolved by X-ray crystallography, with a resolution below 3.6Å and a maximum pair-wise SI of 50%. Since we wanted to create a dataset of RNA-only structures, we used “pdbx_keywords”=“RNA”. We ended up with 117 structures, out of which 88 have an associated RFAM family [15]. These parameters are the same as those used in the construction of the dataset curated in [12], in which only 69 families were included (PDB database accessed in 2020). We note that although there has been an increase in the number of resolved RNA structures over the years, the data remains scarce and significant improvements are needed to train DL models on these limited data.

This dataset labelled with $\mathcal {D_{C}}$ is then used to assess the performance of two unsupervised RNA contact prediction methods, namely PyDCA [19] and Barnacle [20]. In Fig. 1B, we present the performance of these two methods on $\mathcal {D_{C}}$, as measured by the Precision at rank L, which represents the proportion of correctly predicted nucleotide contacts among the top L predictions.

We note that the two methods reach good performances with Precision equal to 0.62 for Barnacle and 0.45 for PyDCA when averaged on all structures belonging to $\mathcal {D_{C}}$. Barnacle [20], which utilizes data-efficient machine learning, generally achieves higher precision than PyDCA, which relies on the pseudo-likelihood maximization direct coupling analysis (DCA) approach as it is more effective in leveraging multiple sequence alignment (MSA) information to predict contacts. Supplementary Table S2 and Supplementary Data contain all the top L precision values for all structures in $\mathcal {D_{C}}$.

There is no clear trend between bit score and precision, as both tools demonstrate variability in performance across different bit scores. This is because not only the bit score but also the effective number of sequences in the RFAM family plays a significant role in the ability of methods to extract structural information from MSA [12].

### Use-case 2: automated RNA structures curation for quick assessment of AlphaFold3 capabilities on RNA

AlphaFold3 [18] shows remarkable promise in protein structure prediction and also promises to predict RNA and RNA complexes. Here, we assess its capabilities to predict the structure of unseen RNA sequences. To conduct such a comprehensive and unbiased assessment, we meticulously crafted two datasets using NucleoSeeker.

Our first dataset, which we designated $\mathcal {D}_{22}$, comprised 213 RNA structures solved before 2023 as the training dataset used by AlphaFold3 was derived from the PDB accessed on January 2023. We applied stringent selection criteria to ensure the highest quality and representativeness of this dataset (for a detailed description of these criteria see Supplementary Data Section S3.2).

Complementing this, we created a second dataset, $\mathcal {D}_{23-24}$, consisting of 27 structures solved in 2023 and 2024. This more recent dataset was important for assessing Alphafold3’s performance on newly determined structures. Since we know, that SI is an important factor in reducing redundancy, we categorize the structures in $\mathcal {D}_{23-24}$ based on their similarity to those in $\mathcal {D}_{22}$ by calculating pairwise SI between all structures across both datasets and dividing the $\mathcal {D}_{23-24}$ entries in three subclasses according to their SI: <50%, between 51%–75%, and >76% (see Supplementary Table S3 and Supplementary Fig. S3).

In Fig. 1C, we plot the pTM score, a confidence metric for the predicted structure from AlphaFold3 [18], against the RMSD between the predicted and experimental structure, for all structures in $\mathcal {D}_{23-24}$ and according to their SI with the closest match in the AF3 training set. Our quick assessment revealed that the AlphaFold3 confidence score genuinely provides a good indication of prediction quality, as high pTM scores correspond to RMSD values generally <5 Å. Additionally, the performance of AF3 improves for structures with higher SI, as indicated by the green diamonds. This observation suggests that the model’s accuracy is influenced by the similarity between the target structure and those in AF3 training data (check Section S3.2 of the Supplementary Data for more details).

While Alphafold3 shows promising performance, especially for RNAs with some degree of similarity to known structures, this preliminary analysis shows that there is still room for improvement in predicting novel or highly divergent RNA structures. As we move forward, these insights will guide our efforts to refine and enhance RNA structure prediction methodologies.

## Performance analysis

We evaluated the performance of NucleoSeeker by comparing the computational performance for generating the two previously discussed datasets ($\mathcal {D}_{22}$ and $\mathcal {D}_{23-24}$). The first dataset ($\mathcal {D}_{22}$) is larger than the second dataset ($\mathcal {D}_{23-24}$) because it is created by considering all structures released till 2022. We assessed the progressive reduction in dataset size at each filtering stage and the computational time required for each step.

The filtering parameters determine the data reduction at each filter level. Starting from the complete PDB RNA structure collection (7704 structures), the Metadata filter typically reduces the dataset by 50%–55%. For $\mathcal {D}_{23-24}$ it retained 3650 structure, while for $\mathcal {D}_{22}$, it retained 206 structures. The IndividualStructure filter further reduced these datasets by 50%–55%, yielding 1547 and 137 structures, respectively.

The computational demands of the pipeline exhibit no surprises and an expected scaling behavior. Tested on some standard hardware (AMD EPYC 7742, 2.25 GHz), the complete curation process required 428 min for the larger dataset, compared to 23 min for the smaller dataset. The structure comparison filter, which represents the most computationally intensive step, exhibited quadratic scaling characteristics, requiring 329 min for processing 1547 structures versus 10 min for 84 structures. This scaling behavior aligns with the theoretical complexity of performing all-against-all structure comparisons.

The final cmscan step showed relatively consistent small processing times for the two datasets. This consistency can be attributed to the effective reduction in dataset size by previous filtering steps, demonstrating how our sequential filtering approach optimizes computational resource utilization by applying more intensive analyses only to high-quality candidates.

These performance characteristics allow to estimate both compute consumption and scaling behavior of our package. For ease of use, the tool also reports the number of structures retained after each filtering step and the corresponding computational time required.

## Discussion

NucleoSeeker targets to complement the development of efficient DL methods for RNA structure prediction, by providing a robust and flexible method for curating high-quality datasets from the PDB database. One of the key strengths of this software is its ability to apply a wide range of filters at both the structure and sequence levels, allowing researchers to create highly specific and relevant datasets tailored to their particular research needs. This functionality is particularly valuable given the challenges associated with RNA data, such as high redundancy, poor resolution, and the presence of hybrid structures. Moreover, the system is designed to ensure that datasets remain up-to-date, even in the rapidly evolving field of RNA research, where new structures are continually being determined and added to the database. Additionally, the modular design of NucleoSeeker allows its components to be used independently or in combination, providing researchers with a high degree of flexibility. Whether the goal is to filter specific structures, analyze polymer chains, or reduce dataset redundancy, it offers the tools needed to achieve these objectives efficiently in a simple way.

## Supplementary Material

lqaf021_Supplemental_File

## Data Availability

The code for NucleoSeeker is available on GitHub at https://github.com/theuutkarsh/nucleoseeker and on Zenodo https://doi.org/10.5281/zenodo.13843170. The package is also available through PyPi, Python Package Index.

## References

[1] LeCun Y, Bengio Y, Hinton G Deep learning. Nature. 2015; 521:436–44. 10.1038/nature14539. 26017442

[2] Jumper J, Evans R, Pritzel A et al. Highly accurate protein structure prediction with AlphaFold. Nature. 2021; 596:583–9. 10.1038/s41586-021-03819-2. 34265844 PMC8371605

[3] Weigt M, White RA, Szurmant H et al. Identification of direct residue contacts in protein–protein interaction by message passing. Proc Natl Acad Sci. 2009; 106:67–72. 10.1073/pnas.0805923106. 19116270 PMC2629192

[4] Schug A, Weigt M, Onuchic JN et al. High-resolution protein complexes from integrating genomic information with molecular simulation. Proc Natl Acad Sci. 2009; 106:22124–9. 10.1073/pnas.0912100106. 20018738 PMC2799721

[5] De Leonardis E, Lutz B, Ratz S et al. Direct-coupling analysis of nucleotide coevolution facilitates RNA secondary and tertiary structure prediction. Nucleic Acids Res. 2015; 43:10444–55. 26420827 10.1093/nar/gkv932 PMC4666395

[6] Pucci F, Schug A Shedding light on the dark matter of the biomolecular structural universe: progress in RNA 3D structure prediction. Methods. Experimental and Computational Techniques for Studying Structural Dynamics and Function of RNA. 2019; 162–163:68–73. 10.1016/j.ymeth.2019.04.012. 31028927

[7] Berman HM, Westbrook J, Feng Z et al. The protein data bank. Nucleic Acids Res. 2000; 28:235–42. 10.1093/nar/28.1.235. 10592235 PMC102472

[8] de Hond AA, Leeuwenberg AM, Hooft L et al. Guidelines and quality criteria for artificial intelligence-based prediction models in healthcare: a scoping review. NPJ Dig Med. 2022; 5:2 10.1038/s41746-021-00549-7. PMC8748878 35013569

[9] Apicella A, Isgrò F, Prevete R Don’t push the button! exploring data leakage risks in machine learning and transfer learning. arXiv20 October 2024, preprint: not peer reviewed https://arxiv.org/abs/2401.13796.

[10] Cruz JA, Blanchet MF, Boniecki M et al. RNA-Puzzles: a CASP-like evaluation of RNA three-dimensional structure prediction. RNA. 2012; 18:610–25. 10.1261/rna.031054.111. 22361291 PMC3312550

[11] Das R, Kretsch RC, Simpkin AJ et al. Assessment of three-dimensional RNA structure prediction in CASP15. Proteins. 2023; 91:1747–70. 10.1002/prot.26602. 37876231 PMC10841292

[12] Pucci F, Zerihun MB, Peter EK et al. Evaluating DCA-based method performances for RNA contact prediction by a well-curated data set. RNA. 2020; 26:794–802. 10.1261/rna.073809.119. 32276988 PMC7297115

[13] Adamczyk B, Antczak M, Szachniuk M RNAsolo: a repository of cleaned PDB-derived RNA 3D structures. Bioinformatics. 2022; 38:3668–70. 10.1093/bioinformatics/btac386. 35674373 PMC9272803

[14] Nawrocki EP, Eddy SR Infernal 1.1: 100-fold faster RNA homology searches. Bioinformatics. 2013; 29:2933–5. 10.1093/bioinformatics/btt509. 24008419 PMC3810854

[15] Kalvari I, Nawrocki EP, Ontiveros-Palacios N et al. Rfam 14: expanded coverage of metagenomic, viral and microRNA families. Nucleic Acids Res. 2021; 49:D192–200. 33211869 10.1093/nar/gkaa1047 PMC7779021

[16] Sievers F, Wilm A, Dineen D et al. Fast, scalable generation of high-quality protein multiple sequence alignments using Clustal Omega. Mol Syst Biol. 2011; 7:539 10.1038/msb.2011.75. 21988835 PMC3261699

[17] Rice P, Longden I, Bleasby A EMBOSS: the european molecular biology open software suite. Trends Genet. 2000; 16:276–77. 10.1016/S0168-9525(00)02024-2. 10827456

[18] Abramson J, Adler J, Dunger J et al. Accurate structure prediction of biomolecular interactions with AlphaFold 3. Nature. 2024; 630:493–500. 10.1038/s41586-024-07487-w. 38718835 PMC11168924

[19] Zerihun MB, Pucci F, Peter EK et al. Pydca v1.0: a comprehensive software for direct coupling analysis of RNA and protein sequences. Bioinformatics. 2020; 36:2264–5. 10.1093/bioinformatics/btz892. 31778142

[20] Taubert O, von der Lehr F, Bazarova A et al. RNA contact prediction by data efficient deep learning. Commun Biol. 2023; 6:1–8. 10.1038/s42003-023-05244-9. 37674020 PMC10482910

[21] JSC JUWELS cluster and booster: exascale pathfinder with modular supercomputing architecture at juelich supercomputing centre. J Large-scale Res Facil. 2021; 7:A138 10.17815/jlsrf-7-183.

[22] JSC JURECA: data centric and booster modules implementing the modular supercomputing architecture at Jülich supercomputing centre. J Large-Scale Res Facil. 2021; 7:10.17815/jlsrf-7-182.

